# Synthesis and Bioassay of a New Class of Furanyl-1,3,4-Oxadiazole Derivatives

**DOI:** 10.3390/molecules18078550

**Published:** 2013-07-19

**Authors:** Mohamed M. El Sadek, Seham Y. Hassan, Huda E. Abdelwahab, Galila A. Yacout

**Affiliations:** 1 Chemistry Department, Faculty of Science, Alexandria University, Alexandria 21231, Egypt; E-Mails: sehamyassen@yahoo.com (S.Y.H.); huda_eid@yahoo.com (H.E.A.); 2 Biochemistry Department, Faculty of Science, Alexandria University, Alexandria 21231, Egypt; E-Mail: galila_69@yahoo.com

**Keywords:** carbohydrazide, oxadiazole, triazole, tyrosinase

## Abstract

Tyrosinase enzyme is a monophenol monoxygenase enzyme, which plays an important role in human as a rate limiting step enzyme for different specific metabolic pathways, as well as its useful application in industry and agriculture. So this study was carried out to test the effect of newly prepared compounds containing 1,3,4-oxadiazoles with different substituted groups on tyrosinase enzyme activity, hoping to use them in the treatment of some diseases arising from tyrosinase activity disorders such as Parkinson’s disease, schizophrenia, autism, attention deficit, hyperactivity disorder, and cancer.

## 1. Introduction

1,3,4-Oxadiazoles are of great practical significance [1] which is primarily due to their large number of uses, in the most diverse areas, for example in drug synthesis, as scintillation materials, in the production of polymers and dyes, and uses in photography as light screening agents. 1,3,4-Oxadiazole (OXD) derivatives are useful targets in the search for antivirals as they have been associated with many types of biological properties such as anti-inflammatory [2,3], antibacterial, antifungal activities [4,5] and HIV replication inhibition [6]. Oxadiazoles have often been described as bio-isosteres for amides and esters [7]. As a consequence of these characteristics, oxadiazoles have impacted drug discovery programs in numerous areas including muscarinic agonists [8,9], benzodiazepine receptor partial agonists [10,11], dopamine transporters [12], anti-rhinovirals [13], growth hormone secretogogues [14], 5-HT agonists[15], antispasmodics [16], nematocidal, fungicidal and microbicides [17], analgesics [18], anti-inflammatory agents [19,20], Fab I inhibitors as antibacterial agents [21], immnosuppressants [22], and also antiplatelet and antithrombotic agents [23], 5-HT antagonists [24], human NK_1_ antagonists [25], They have been used as peptide mimetics due to their particular geometric and electrostatic properties [26,27]. Accordingly, in continuation of our work [28,29,30,31,32,33,34], a variety of heterocyclic derivatives involving some new oxadiazoles have been prepared from saccharide derivatives, and their chemistry and the effect of these derivatives on tyrosinase enzyme [35,36,37,38], which is the rate limiting step in melanine biosynthesis [39] as well as different biological actions were studied [40].

## 2. Results and Discussion

### 2.1. Chemistry

**Scheme 1:**
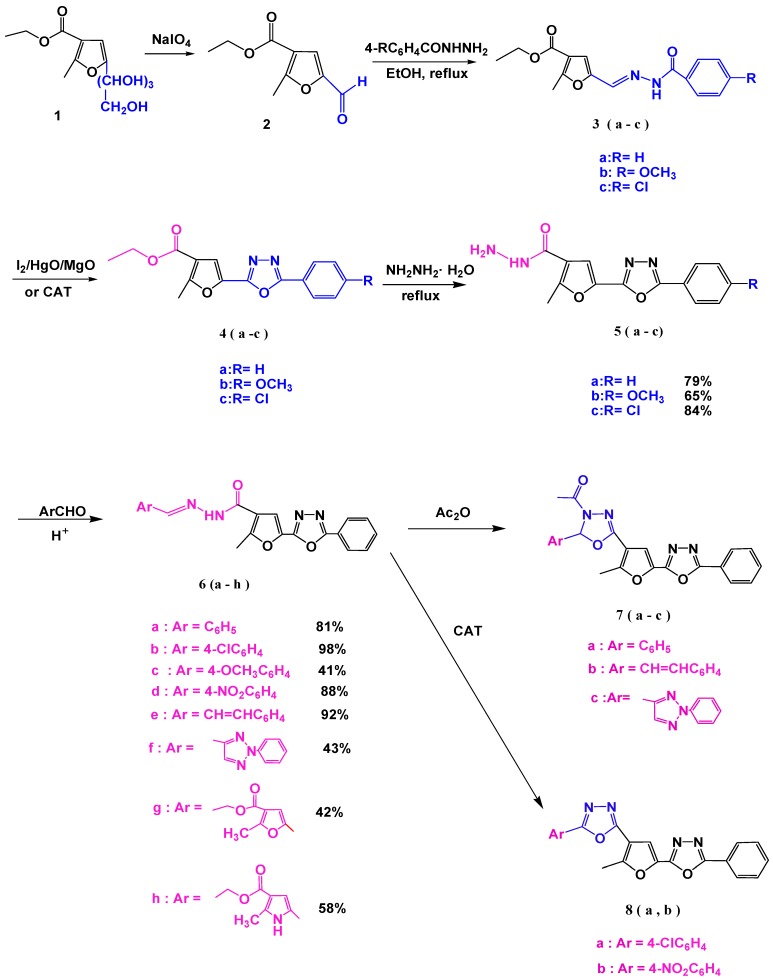
Synthesis of 1,3,4-oxadiazole derivatives.

Ethyl 5-(1,2,3,4-tetrahydroxybutyl)-2-methylfuran-3-carboxylate(**1**) [41]was oxidized to the corresponding formyl derivative **2** [42,43], which was condensed with a number of aroylhydrazines to afford hydrazones **3a–c** [44]. Oxidative cyclization of compounds **3a–c** with iodine, yellow mercuric oxide, and magnesium oxide in dry ether [44] (yields 41%–47%) or by an improved procedure through refluxing with chloramine-T in ethanol [32] (which gave higher yields: 78%–91%) afforded the corresponding 1,3,4-oxadiazole derivatives **4a–c**. Furthermore, boiling 1,3,4-oxadiazole derivatives **4a–c** with hydrazine hydrate afforded the corresponding hydrazide derivatives, the 2-methyl-5-(5-phenyl-1,3,4-oxadiazol-2-yl)furan-3-carbohydrazides **5a–c** (Scheme 1).

The ^1^H-NMR spectra of compounds **5a–c** showed the disappearance of both the CH_3_ and CH_2_ protons of the ethyl ester group. Instead, they displayed two signals for the NH_2_protons in the δ 4.59–1.64range, and the NH proton of the hydrazide group at 9.82–9.00 ppm, respectively (for other protons see Experimental). The mass spectra of compounds **5a** and **5c** showed the molecular ion peaks at *m/z* 284 and 318, respectively. The base peaks of compound **5a** and **5c** appeared at *m/z* 171 and 105, respectively.

On the other hand, condensation of the hydrazide derivative **5a** with a number of aldehydes afforded the corresponding hydrazone derivatives **6a–h** (Scheme 1). The^1^H-NMR spectra of compounds **6a–h** showed the disappearance of the NH_2_ protons and instead, displayed a singlet signal for the CH=N protons in the δ 10.2–8.30 range, while the NH protons appeared as a singlet at δ 8.85, 9.98, 7.85, 8.80, 9.52, and 9.38, respectively (see Experimental). The mass spectra of the compounds **6d**, **6e**, **6f** and **6h** showed the molecular ion peaks at *m/z* 417, 393,440 and 447, respectively; while the base peaks appeared at *m/z* 176, 115, 77 and 358, respectively (see Experimental).

Cyclization of the hydrazones **6a**, **6e**, and **6f** with acetic anhydride under reflux, afforded the oxadiazoline derivatives **7a–c** (Scheme 1). The mechanism of their formation probably proceeded through the highly stable enolized form.

The ^1^H-NMR spectra of compounds **7a–c** showed the disappearance of both the NH and CH=N protons. Instead, their ^1^H-NMR showed the methyl protons of the N-COCH_3_ group as a singlet in the δ 1.24–1.22 ppm range (see Experimental). In addition, the proton at position-5 in the triazole ring (Compound **7c**) resonates at lower field than the proton of the oxadiazolyl ring due to the strong electron attracting property of the triazole moiety. On the other hand, oxidative cyclization of the hydrazone compounds **6b** and **6d** with chloramine-T afforded the corresponding 1,3,4-oxadiazolederivatives **8a** and **8b** in high yield (Scheme 1). The ^1^H-NMR spectra of compounds **8a** and **8b** showed the disappearance of both the NH and (CH=N protons (see Experimental). The mass spectrum of compound **8b** showed the expected molecular ion peak at *m/z* 415, while the base peak appeared at *m/z* 91.

### 2.2. Biological Activity Assays

#### 2.2.1. Enzyme Activity Assay

Tyrosinase enzyme was prepared from mushrooms in a phosphate buffer (50 mM, pH 6.0) according to the method of Yang and Robb [45]. The activity of the prepared enzyme solution was determined by following spectrophotometrically the formation of dopachrome at 30 °C. After addition of enzyme preparation (50 μL) to a cuvette containing phosphate buffer (1.2 mL, 50 mM, pH 6.0) and 10 mM L-dopa (0.8 mL), the solution was immediately mixed and the increase in absorbance at 475 nm (indicating the formation of dopachrome) was recorded with a UV-20100 spectrophotometer. A blank experiment was carried out as mentioned above using buffer (50 μL) instead of enzyme preparation [46].

#### 2.2.2. Enzyme Activity Assay in the Presence of the Tested Compounds

**Figure 1:**
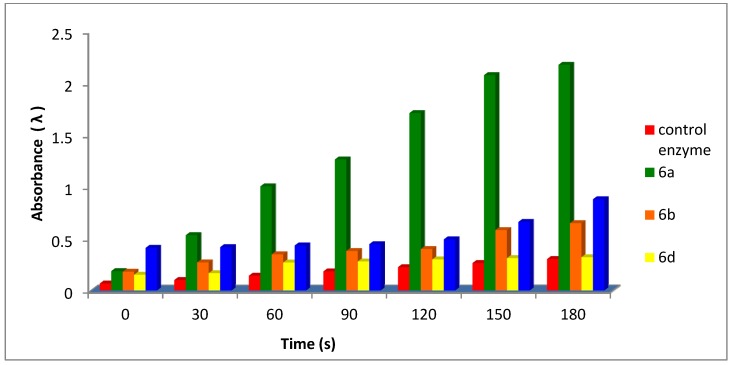
Effect of time (s) on the absorbance of tyrosinase-catalyzed reaction in presence of the examined compounds **6a**, **6b**, **6d** and **6g** compared to control enzyme.

**Figure 2:**
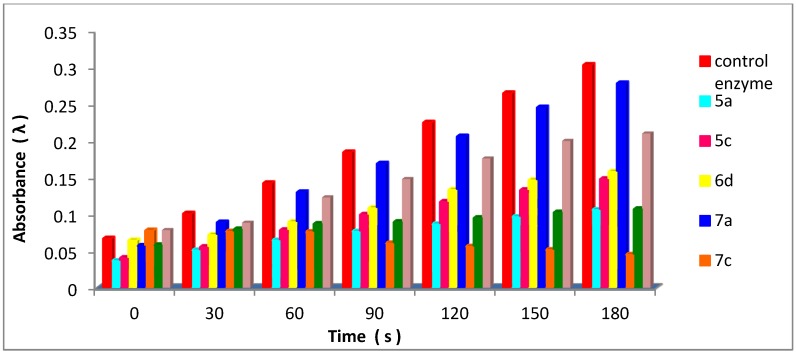
Effect of time (s) on the absorbance of tyrosinase-catalyzed reaction in presence of the examined compounds **5a**, **5c**, **6f**, **7a**, **7c**, **8a**, **8b** compared to control enzyme.

Activity of the enzyme in the presence of the examined compounds was determined by following the above steps for the formation of dopachrome and each examined compound (0.8 mL, 10 mmol) separately, and the increase in absorbance at 475 nm was recorded, separately, as shown in Table 1 and Table 2 and Figure 1 and Figure 2. All tests carried out in triplicate.

**Table 1:** Effect of time (s) the absorbance of on tyrosinase-catalyzed reaction in presence of the examined compounds **6a**, **6b**, **6d** and **6g** compared to control enzyme.

Time (s)	Absorbance (λ)				
	Control enzyme	6a	6b	6d	6g
0	0.0670	0.1870	0.1810	0.1520	0.4115
30	0.1010	0.5340	0.2710	0.1670	0.4185
60	0.1425	1.0050	0.3490	0.2700	0.4345
90	0.1845	1.2650	0.3810	0.2800	0.4470
120	0.2250	1.7130	0.4000	0.3000	0.4935
150	0.2650	2.0790	0.5830	0.3120	0.6620
180	0.3035	2.1790	0.6500	0.3210	0.8810

**Table 2:** Effect of time (s) on the absorbance of tyrosinase-catalyzed reaction in presence of the examined compounds **5a**, **5c**, **6f**, **7a**, **7c**, **8a**, **8b** compared to control enzyme.

Time (s)	Absorbance (λ)							
	Control enzyme	5a	5c	6f	7a	7c	8a	8b
0	0.0670	0.0365	0.0405	0.0645	0.0570	0.0780	0.0580	0.0785
30	0.1010	0.0510	0.0555	0.0720	0.0890	0.0765	0.0795	0.0885
60	0.1425	0.0645	0.0785	0.0895	0.1300	0.0760	0.0870	0.1230
90	0.1845	0.0765	0.0995	0.1085	0.1790	0.0605	0.0895	0.1480
120	0.2250	0.0865	0.1170	0.1334	0.2060	0.0560	0.0950	0.1760
150	0.2650	0.0965	0.1330	0.1465	0.2455	0.0515	0.1025	0.2000
180	0.3035	0.1060	0.1480	0.1580	0.2785	0.0450	0.1070	0.2100

### 2.3. Results

Our obtained data revealed that the examined compounds showed different effects [47] on the tyrosinase enzyme activity between inhibition and activation in which compounds containing one 1,3,4-oxadiazole ring in addition to the furan ring (compounds **6a**, **6b**, **6d**, **6g**) have an activating effect on the enzyme tyrosinase with different values(Table 1 and Figure 1). Compound **6a** in which there is no substituent on the phenyl group, which probably makes it easier to bind with the active sites of the enzyme, showed the highest activation effect. The presences of substituents on the phenyl group such as halogens, or a nitro group (compounds **6b**, **6d**) decrease the activation ability of the compound, while compounds containing a CONHNH_2_ group have inhibitory effects (**5a**, **5c**).

On the other hand compounds containing two 1,3,4-oxadiazole rings or one oxadiazole ring and one 1,2,3-triazole ring have inhibitory effects on tyrosinase enzyme (compounds **6f**, **7a**, **8a** and **8b**). In addition compound **7c** which contains two 1,3,4-oxadiazoles and one 1,2,3-triazole ring has the highest inhibition effect (Table 2 and Figure 2). The increasing number of heterocyclic rings containing nitrogen such as 1,3,4-oxadiazole and 1,2,3-triazole rings has a noticeable inhibitory effect on tyrosinase enzyme.

## 3. Experimental

### 3.1. General Methods

Melting points were determined on a Köfler block and are uncorrected. IR spectra were recorded on a Perkin Elmer 1600 spectrometer. ^1^H-NMR was recorded on a JEOL JNM ECA 500 MHz instrument using tetramethylsilane as an internal standard. Mass spectra were recorded on a GC-MS solution DI Analysis Shimadzu Qp-2010 unit. Elemental analysis was determined at the Regional Center for Mycology and Biotechnology, Al-Azhar University. Thin layer chromatography (TLC) was carried out on silica gel plates. Solutions were evaporated under diminished pressure unless otherwise stated. The ChemDrew-Ultra-8.0 software was used for naming the prepared compounds.

### 3.2. Reactions of Compounds **4a–c** with Hydrazine Hydrate

A mixture of ethyl-5-(5-(4-substituted phenyl)-1,3,4-oxadiazole-2-yl)-2-methylfuran-3-carboxylates **4a–c** (1 g, 3.4 mmol), and hydrazine hydrate (5 mL, 103 mmol) was refluxed for one hour. The resulting solution was left at R.T. for one hour, and the product that separated out was filtered off, washed with a little ethanol, recrystallized from ethanol and dried.

*2-Methyl-5-(5-phenyl-1,3,4-oxadiazol-2-yl) furan-3-carbohydrazide* (**5a**). Yield: 682 mg (79%). White crystals; m.p. 103–104 °C; R_f_: 0.72 (*n*-hexane/EtOAc, 3:1, V/V); IR (KBr): 1,621 (C=N), 1,643 (C=O amide), 3,142 (NH), 3,321 cm^−1^ (NH_2_); ^1^H-NMR (CDCl_3_) δ: 1.64 (bs, 2*H*, NH_2_; exchangeable with D_2_O), 2.73 (s, 3*H*, CH_3_), 7.45 (s,1*H*, H-furan), phenyl protons: 7.50–7.56 (m, 3*H*, *m*-H, *p*-H), 8.10–8.13 (dd, 2*H*, *o*-H; *J*_1,2_ = 2.3 Hz, *J*_1,3_ = 6.9 Hz), 9.00 (s, 1*H*, NH; exchangeable with D_2_O); MS: *m/z* (%), 51 (10.85), 63 (13.22), 64 (7.87), 65 (46.02), 77 (17.44), 79 (5.49), 80 (4.51), 89 (13.57), 90 (7.27), 91 (80.36), 92 (12.96), 93 (80.36), 105 (14.12), 106 (16.08), 107 (51.20), 108 (30.53), 155 (70.23), 156 (6.30), 167 (0.17), 171 (100), 172 (9.42), 284 (5.47, M^+^); Anal. Calcd for C_14_H_12_N_4_O_3_ (284.27): C, 59.15; H, 4.25; N, 19.71; Found: C, 58.94; H, 4.09; N, 19.59.

*5-(5-(4-Methoxyphenyl)-1,3,4-oxadiazol-2-yl)-2-methylfuran-3-carbohydrazide* (**5b**). Yield: 620 mg (65%). White crystals; m.p. 121–122 °C; R_f_: 0.86 (CHCl_3_/MeOH, 25:1, V/V); IR (KBr): 1,599 (C=N), 1,656 (C=O amide), 3,261 (NH), 3,360cm^−1^ (NH_2_); ^1^H-NMR(DMSO-*d*_6_,) δ: 2.24 (s, 3*H*, CH_3_-furan), 3.88 (s, 3*H*, OCH_3_), 4.59 (bs, 2*H*, NH_2_; exchangeable with D_2_O), 7.23 (s, 1*H*, H-furan), 7.47 (d, 2*H*, *o*-OCH3; *J* = 8.4 Hz), 7.80 (d, 2*H*, *m*-OCH3; *J* = 8.4 Hz), 9.82 (bs, 1*H*, NH; exchangeable with D_2_O); Anal. Calcd for C_15_H_14_N_4_O_4_ (314.3): C, 57.32; H, 4.49; N, 17.83; Found: C, 57.16; H, 4.37; N, 17.78.

*5-(5-(4-Chlorophenyl)-1,3,4-oxadiazol-2-yl)-2-methylfuran-3-carbohydrazide* (**5c**). Yield: 810 mg (84%). White crystals; m.p. 153–154 °C; R_f_: 0.77 (CHCl_3_/MeOH, 25:1, V/V); IR (KBr): 1,618 (C=N), 1,661 (C=O amide), 3,224, 3,310 cm^−1^ (NH, NH_2_); ^1^H-NMR (DMSO-*d*_6_) δ: 2.24 (s, 3*H*, CH_3_-furan), 4.59 (bs, 2*H*, NH_2_; exchangeable with D_2_O), 7.23 (s, 1*H*, H-furan), 7.48 (d, 2*H*, *o*-Cl; *J* = 8.4 Hz), 7.79 (d, 2*H*, *m*-Cl; *J* = 8.4 Hz), 9.82 (bs, 1*H*, NH; exchangeable with D_2_O); MS: *m/z* (%), 50 (15.66), 51 (16.66), 55 (24.81), 56 (13.73), 57 (32.84), 60 (15.93), 65 (15.33), 69 (18.74), 71 (15.62), 75 (14.92), 77 (45.62), 83 (15.34), 85 (11.56), 91 (35.71), 97 (12.70), 105 (100), 106 (13.09), 107 (11.69), 111 (51.13), 113 (10.93), 138 (11.03), 139 (33.40), 141 (11.32), 155 (10.76), 171 (12.76), 309 (11.93), 318 (11.93, M^+^); Anal. Calcd for C_14_H_11_ClN_4_O_3_ (318.72): C, 52.76; H, 3.48; N, 17.58; Found: C, 52.89; H, 3.51; N, 17.43.

### 3.3. Reactions of Carbohydrazide **5a** with Aldehydes

A solution of 2-methyl-5-5-phenyl-1,3,4-oxadiazole-2-l)furan-3-carbohydrazide (**5a**, 500 mg, 1.8 mmol) in ethanol (15 mL, 257 mmol) containing acetic acid (0.1 mL, 1.75 mmol) was treated with aldehyde (1.8 mmol) in ethanol (10 mL, 171 mmol). The mixture was refluxed on a water bath for 10 min, and after cooling, the product that separated out was filtered off, washed with little ethanol, recrystallized from ethanol and dried.

*N-Benylidene-2-methyl-5-(5-phenyl-1,3,4-oxadiazole-2-yl)furan-3-carbohydrazide* (**6a**). Yield: 317 mg (81%). Pale yellow crystals; m.p. 73–74 °C; R_f_: 0.42 (*n*-hexane/EtOAc, 7:1, V/V); IR (KBr): 1,559 (C=N), 1,641 (C=O amide), 3,320 cm^−1^ (NH); ^1^H-NMR (CDCl_3_) δ: 2.76 (s, 3H, CH_3_-furan), 7.43 (s, 1*H*, H-furan), 7.53–7.57 (m, 4*H*, Ar-H), 7.62–7.66 (m, 1*H*, Ar-H), 7.89 (d, 2*H*, Ar-H; *J* = 6.9 Hz), 8.11–8.14 (m, 3*H*, Ar-H), 8.85 (s, 1*H*, NH; exchangeable with D_2_O), 10.02 (s, 1*H*, CH=N). Anal. Calcd for C_21_H_16_N_4_O_3_ (372.38): C, 67.73; H, 4.33; N, 15.05; Found: C, 67.64; H, 4.32; N, 14.93.

*N-(4-Chlorobenzyldine)-2-methyl-5-(5-phenyl-1,3,4-oxadiazole-2-yl)furan-3-carbohydrazide* (**6b**). Yield: 760 mg (98%). Faint golden crystals; m.p. 198–199 °C; R_f_: 0.91 (*n*-hexane/EtOAc, 7:1, V/V); IR (KBr): 1,623 (C=N), 1,655 (C=O amide), 3,241 cm^−1^ (NH), ^1^H-NMR (CDCl_3_) δ: 2.21 (s, 3*H*, CH_3_-furan), 7.26 (s, 1*H*, H-furan), 7.34–7.38 (m, 2*H*, Ar-H), 7.44 (d, 2*H*, *o*-Cl; *J* = 8.4Hz), 7.51–7.53 (m, 2*H*, Ar-H), 7.81 (d, 2*H*, *m*-Cl; *J* = 8.4 Hz), 8.04 (d, 1*H*, Ar-H; *J* = 8.4 Hz), 8.67 (s, 1*H*, CH=N), 9.98 (s, 1*H*, NH; exchangeable with D_2_O); Anal. Calcd for C_21_H_15_ClN_4_O_3_ (406.82): C, 62.00; H, 3.72; N, 13.77; Found: C, 61.89; H, 3.58; N, 13.73.

*N'-(4-Methoxybenzylidene)-2-methyl-5-(5-phenyl-1,3,4-oxadiazol-2-yl)furan-3-carbohydrazide* (**6c**). Yield: 580 mg (41%). Yellow crystals; m.p. 166–167 °C; R_f_: 0.41 (*n*-hexane/EtOAc, 7:1, V/V); IR (KBr): 1,602 (C=N), 1,642 (C=O amide), 3,176 cm^−1^ (NH); Anal. Calcd for C_22_H_18_N_4_O_4_ (402.4): C, 65.66; H, 4.51; N, 13.92; Found: C, 65.44; H, 4.34; N, 14.00.

*N-(4-Nitrobenzylidene)-2-methyl-5-(5-phenyl-1,3,4-oxadiazol-2-yl)furan-3-carbohydrazide* (**6d**). Yield: 640 mg (88%). Golden crystals; m.p. 295–296°C; R_f_: 0.56 (*n*-hexane/EtOAc, 7:1, V/V); IR (KBr): 1,596 (C=N), 1,652 (C=O amide), 3,431cm^−1^ (NH), ^1^H-NMR (CDCl_3_); δ: 2.16 (s, 3*H*, CH_3_-furan), 6.75 (s, 1*H*, H-furan), 7.54–7.57 (m, 1*H*, Ar-H), 7.85 (bs, 1*H*, NH; exchangeable with D_2_O), 8.02–8.14 (m, 4*H*, Ar-H), 8.27–8.40 (m, 4*H*, Ar-H), 8.71 (s, 1*H*, CH=N); MS: *m/z* (%), 50 (37.75), 51 (21.53), 63 (28.45), 64 (9.55), 76 (66.32), 77 (25.51), 89 (17.28), 91 (6.32), 92 (6.75), 102 (8.13), 103 (39.92), 104 (13.76), 118 (6.65), 130 (41.48), 149 (9.77), 150 (7.10), 151 (8.98), 152 (5.50), 165 (2.84), 166 (2.89), 167 (2.20), 176 (100), 177 (13.94), 178 (12.27), 193 (3.50), 205 (11.20), 206 (6.51), 222 (1.81), 251 (21.90), 252 (5.49), 271 (7.56), 281 (2.09), 297 (24.10), 298 (54.66), 299 (9.33), 417 (11.21, M^+^). Anal. Calcd for C_21_H_15_N_5_O_5_ (417.37): 417.37; C, 60.43; H, 3.62; N, 16.78; Found: C, 60.34; H, 3.57; N, 16.67.

*2-Methyl-5-(5-phenyl-1,3,4-oxadiazole-2-yl)-N-(3-phenylallylidene)furn-3-carbohydrazide* (**6e**). Yield: 513 mg (91%). Yellow crystals; m.p. 158–159 °C; R_f_: 0.61 (*n*-hexane/EtOAc, 7:1, V/V); IR (KBr): 1559 (C=N), 1,641 (C=O amide), 3,301cm^−1^ (NH), ^1^H-NMR (CDCl_3_) δ: 2.72 (s, 3*H*, CH_3_-furan), 7.13–7.21 (m, 1*H*, Ph-CH), 7.19 (s, 1*H*, H-furan), 7.35–7.57 (m, 10*H*, Ar-H), 8.03–8.15 (m, 1*H*, CH=), 8.46 (d, 1*H*, CH=N; *J*= 8.4 Hz), 8.80 (bs, 1*H*, NH; exchangeable with D_2_O); MS: *m/z* (%), 50 (5.66), 51 (19.21), 52 (4.67), 63 (9.60), 65 (5.70), 77 (50.41), 78 (10.66), 89 (11.09), 91 (69.20), 102 (16.88), 103 (42.06), 104 (16.28), 115 (100), 116 (17.95), 117 (8.46), 129 (40.84), 130 (95.23), 131 (11.37), 142 (2.91), 156 (20.45), 157 (12.07), 183 (7.91), 206 (2.88), 217 (4.28), 232 (76.86), 233 (58.29), 234 (9.82), 259 (64.74), 260 (55.93), 261 (11.03), 395 (2.13,M^+^-3); Anal. Calcd for C_23_H_18_N_4_O_3_ (398.41): C, 69.34; H, 4.55; N, 14.06; Found: C, 69.11; H, 4.52; N, 14.10.

*2-Methyl-5-(5-phenyl-1,3,4-oxadiazol-2-yl)-N-((2-phenyl-2H-1,2,3-triazole-4-yl)methylene)furan-3-carbohydrazide* (**6f**). Yield: 658 mg (43%). Pale yellow crystals; m.p. 219–220 °C; R_f_: 0.43 (*n*-hexane/EtOAc, 7:1, V/V); IR (KBr): 1,599 (C=N), 1,637 (C=O amide), 3,118 cm^−1^ (NH);^1^H-NMR (DMSO-*d*_6_) δ: 2.32 (s, 3*H*, CH_3_-furan), 7.32 (s, 1*H*, H-furan), 7.42–7.43 (m, 5*H*, Ar-H), 7.91–7.92 (m, 5*H*, Ar-H), 8.96 (s, 1*H*, CH=N), 9.21 (s, 1*H*, NH; exchangeable with D_2_O), 10.42 (s, 1*H*, H-triazole); MS: *m/z* (%), 50 (3.91), 51 (25.87), 59 (0.11), 64 (26.99), 65 (21.02), 77 (100), 91 (84.82), 92 (23.24), 93 (13.51), 104 (25.71), 105 (13.50), 118 (17.28), 128 (2.22), 145 (5.62), 146 (4.82), 158 (4.98), 174 (85.19), 175 (44.51), 184 (5.98), 187 (5.06), 188 (4.33), 209 (3.02), 287 (3.78), 213 (10.36), 342 (37.45), 343 (10.63), 422 (2.47), 439 (5.54, M^+^). Anal. Calcd for C_23_H_17_N_7_O_3_ (439.43): C, 62.87; H, 3.90; N, 22.31; Found: C, 62.61; H, 3.68; N, 22.13.

*Ethyl-5-((2-methyl-5-(5-phenyl-1,3,4-oxadiazole-2-yl)furan-3-carboylimino)methyl)-2-methylfuran-3-carboxylate* (**6g**). Yield: 330 mg (42%). Pale green crystals; m.p. 185–186 °C; R_f_: 0.38 (*n*-hexane/EtOAc, 7:1, V/V); IR (KBr): 1,591 (C=N), 1,643 (C=O amide), 1,707 (CO ester), 3,137cm^−1^ (NH), ^1^H-NMR (DMSO-*d*_6_) δ: 1.35 (t, 3*H*, CH_3_-ester; *J* = 6.9Hz), 2.70 (s, 6*H*, 2CH_3_-furan), 4.31 (q, 2*H*, CH_2_-ester; *J* = 6.9Hz), 7.26 (s, 1*H*, H-furan_1_), 7.45 (s, 1*H*, H-furan_2_), 7.50–7.56 (m, 3*H*, Ar-H), 8.12 (dd, 2*H*, Ar-H; *J*_1,2_ = 2.3 Hz, *J*_1,3_ = 6.9 Hz), 8.48 (s, 1*H*, CH=N), 9.52 (s,1*H*, NH; exchangeable with D_2_O); Anal. Calcd for C_23_H_20_N_4_O_6_ (448.43): C, 61.60; H, 4.50; N, 12.49; Found: C, 61.49; H, 4.44; N, 12.34.

*Ethyl-5-((2-methyl-5-(5-phenyl-1,3,4-oxadiazole-2-yl)furan-3-carboylimino)methyl)-2-methyl-1H-pyrrole-3-carboxylate* (**6h**). Yield: 140 mg (58%). Yellow crystals; m.p. 262–263 °C; R_f_: 0.5 (*n*-hexane/EtOAc, 7:1, V/V); IR (KBr): 1,598 (C=N), 1,656 (C=O amide), 1,675 (CO ester), 2,976 (NH), 3,314 cm^−1^ (NH-pyrrole); ^1^H-NMR (DMSO-*d*_6_) δ: 1.22 (t, 3*H*, CH_3_-ester; *J* = 6.9Hz), 2.42 (s, 3*H*, CH_3_-pyrrol), 2.46 (s, 3*H*, CH_3_-furan), 4.13 (q, 2*H*, CH_2_-ester; *J* = 6.9 Hz), 6.76 (s, 1*H*, H-furan), 6.91 (d, 1*H*, Ar-H; *J* = 2.3 Hz), 7.27 (s, 1*H*, H-pyrrole), 7.60–7.62 (m, 2*H*, Ar-H), 8.02–8.07 (m, 2*H*, Ar-H), 8.30 (s, 1*H*, CH=N), 9.38 (s,1*H*, NHCO; exchangeable with D_2_O), 12.08 (s, 1*H*, NH-pyrrole; exchangeable with D_2_O); MS: *m/z* (%), 66 (3.78), 78 (4.11), 80 (4.40), 106 (4.95), 107 (12.50), 120 (2.26), 124 (1.77), 134 (21.63), 135 (22.36), 136 (4.07), 151 (39.81), 152 (15.73), 165 (5.69), 180 (24.27), 184 (2.00), 228 (2.82), 256 (2.24), 283 (2.13), 313 (14.14), 358 (100), 359 (22.22), 447 (4.95, M^+^). Anal. Calcd for C_23_H_21_N_5_O_5_ (447.44): C, 61.74; H, 4.73; N, 15.65; Found: C, 61.69; H, 4.70; N, 15.76.

### 3.4. Reaction of Compounds **6a**, **6e**, **6f** with Acetic Anhydride

A mixture of **6a**, **6e** or **6f** (100 mg, 27 mmol) and acetic anhydride (1 mL, 10.6 mmol) was refluxed for 15 min on gentle heating. The hot solution was poured onto ice water (10 mL) and the product which separated was filtered off, washed several times with water, recrystallized from ethanol and dried.

*1-(5-(2-Methyl-5-(5-phenyl-1,3,4-oxadiazol-2-yl)furan-3-yl)-2-phenyl-1,3,4-oxadiazol-3(2H)-yl)ethanone* (**7a**). Yield: 92 mg (84%). White powder; m.p. 97–98 °C; R_f_: 0.43(*n*-hexane/EtOAc, 5:1, V/V); IR (KBr): 1,584 (C=N), 1,671 cm^−1^ (C=O acetyl); ^1^H-NMR (CDCl_3_) δ: 1.24 (s, 3*H*, CH_3_CO), 2.85 (s, 3*H*, CH_3_-furan), 7.18 (s, 1*H*, H-furan), 7.23 (s, 1*H*, H-oxadiazoline), 7.36–7.57 (m, 10*H*, 2Ph). Anal. Calcd for C_23_H_18_N_4_O_4_ (414.41): C, 66.66; H, 4.38; N, 13.52; Found: C, 66.90; H, 4.28; N, 13.55.

*1-(5-(2-Methyl-5-(5-phenyl-1,3,4-oxadiazole-2-yl)furan-3-yl)-2-styryl-1,3,4-oxadiazol-3(2H)-yl)ethanone* (**7b**). Yield: 102 mg (98%). Yellow powder; m.p. 150–151 °C; R_f_: 0.67(*n*-hexane/EtOAc, 6:1, V/V); IR (KBr): 1,588 (C=N), 1,669 cm^−1^ (C=O acetyl); ^1^H-NMR (CDCl_3_) δ: 1.24 (s, 3*H*, CH_3_CO), 2.73 (s, 3*H*, CH_3_-furan), 6.34 (s, 1*H*, H-furan), 7.12 (s, 1*H*, H-oxadiazoline), 7.08–7.15 (m, 1*H*, PhCH=), 7.33–7.42 (m, 6*H*, Ar-H), 7.49 (d, 4*H*, Ar-H; *J* = 6.1 Hz), 8.40 (dd, 1*H*, CH=; *J*_1,2_ = 1.6 Hz,*J*_1,3_ = 6.9 Hz); MS: *m/z* (%), 50 (6.82), 51 (22.37), 63 (14.07), 77 (53.47), 91 (49.70), 103 (41.24), 115 (57.27), 128 (10.49), 129 (13.26), 130 (100), 142 (2.54), 156 (36.13), 183 (6.91), 205 (1.96), 217 (2.68), 233 (44.00), 259 (45.70), 260 (90.95), 286 (2.31), 390 (0.02), 397 (0.03), 399 (0.12), 414 (0.12), 434 (0.05), 440 (14.07, M^+^); Anal. Calcd for C_25_H_20_N_4_O_4_ (440.45): C, 68.17; H, 4.58; N, 12.72; Found: C, 68.39; H, 4.44; N, 12.66.

*1-(5-(2-Methyl-5-(5-phenyl-1,3,4-oxadiazol-2-yl)furan-3-yl)-2-(2-phenyl-2H-1,2,3-triazol-4-yl)-1,3,4-oxadiazol-3(2H)-yl)ethanone* (**7c**). Yield: 360mg (33%). White crystals; m.p. 169–170 °C; R_f_: 0.58 (*n*-hexane/EtOAc, 6:1, V/V); IR (KBr): 1,598 (C=N), 1,687cm^−1^ (C=O acetyl);^1^H-NMR (DMSO-*d*_6_) δ: 1.22 (s, 3*H*, CH_3_CO), 1.25 (s, 3*H*, CH_3_-furan), 7.33–7.46 (m, 10 *H*, Ar-H),8.23 (s, 1*H*, H-furan), 8.24 (s, 1*H*, H-oxadiazoline), 9.12 (s, 1*H*, H-triazole); MS: *m/z* (%), 50 (3.35), 51 (23.27), 64 (25.65), 77 (100), 91 (83.64), 104 (30.74), 105 (14.66), 118 (18.87), 128 (2.50), 145 (6.44), 157 (2.35), 170 (17.37), 171 (16.02), 172 (15.57), 184 (7.88), 209 (4.49), 229 (2.02), 247 (0.93), 287 (4.10), 299 (0.28), 311 (0.49), 313 (14.20), 342 (58.26), 428 (13.25), 481 (13.26, M^+^). Anal. Calcd for C_25_H_19_N_7_O_4_ (481.46): C, 62.37; H, 3.98; N, 20.36; Found: C, 62.28; H, 4.20; N, 20.29.

### 3.5. Reaction of Compounds **8a**, **8b** with Chloramine-T

A mixture of **8a** or **8b** (1.28 g, 3.2 mmol) and chloramine-T (710 mg, 3.2 mol) in isopropyl alcohol (50 mL, 654 mmol) was refluxed for 4 hours, then it was concentrated, and after cooling, the product which separated out was filtered off, washed with a little ethanol, recrystallized from ethanol and dried.

*2-(4-(5-(4-Chlorophenyl)-1,3,4-oxadiazol-2-yl)-5-methylfuran-2-yl)-5-phenyl-1,3,4-oxadiazole* (**8a**). Yield: 830 mg (65%). Brown crystals; m.p. 178–179 °C, R_f_: 0.12 (*n*-hexane/EtOAc, 5:1, V/V); IR (KBr): 1,593 cm^−1^ (C=N); ^1^H-NMR (DMSO-*d*_6_) δ: 2.32 (s, 3*H*, CH_3_-furan), 6.87 (s, 1*H*, H-furan), 7.42–7.44 (m, 5*H*, Ar-H), 7.91–7.97 (m, 4*H*, Ar-H). Anal. Calcd forC_21_H_13_ClN_4_O_3_ (404.81): C, 62.31; H, 3.24; N, 13.84; Found: C, 62.11; H, 3.08; N, 13.62.

*2-(5-Methyl-4-(5-(4-nitrophenyl)-1,3,4-oxadiazol-2-yl)furan-2-yl)-5-phenyl-1,3,4-oxadiazole* (**8b**). Yield: 590 mg (81%). It was recrystallized from ethanol as deep yellow crystals; m.p. 97–98 °C; R_f_: 0.13 (*n*-hexane/EtOAc, 5:1, V/V); IR (KBr): 1,597 cm^−1^ (C=N); ^1^H-NMR (DMSO-*d*_6_) δ: 2.34 (s, 3*H*, CH_3_-furan), 7.38 (s, 1*H*, H-furan), 7.43–7.44 (m, 5*H*, Ar-H), 7.92–7.94 (m, 4*H*, Ar-H); MS: *m/z* (%), 57 (5.50), 65 (17.99), 68 (1.60), 69 (5.37), 77 (7.26), 91 (100), 92 (10.33), 93 (3.44), 105 (5.03), 107 (24.57), 108 (18.05), 109 (2.98), 115 (3.92), 121 (2.74), 129 (5.44), 130 (5.60), 149 (25.33), 155 (36.75), 171 (52.53), 183 (2.31), 206 (2.18), 236 (2.61), 252 (1.69), 273 (1.77), 281 (2.21), 319 (1.87), 362 (1.84), 415 (1.82, M^+^). Anal. Calcd for C_21_H_13_N_5_O_5_ (415.36): C, 60.72; H, 3.15; N, 16.86; Found: C, 60.59; H, 2.99; N, 16.69.

## 4. Conclusions

Some new oxadiazole derivatives have been prepared from carbohydrate precursors. Their physical and chemical properties were studied, as well as their effect on the enzyme tyrosinase.
